# Exploring Prototype Networks for Surgical Vision: Interpretability and Performance in Semantic Segmentation and Surgical Phase Recognition

**DOI:** 10.3390/jimaging12080374

**Published:** 2026-08-11

**Authors:** Yiping Li, Ronald L. P. D. de Jong, Franco Badaloni, Gino M. Kuiper, Romy C. van Jaarsveld, Jelle P. Ruurda, Marcel Breeuwer

**Affiliations:** 1 Department of Biomedical Engineering, Eindhoven University of Technology, 5612 AP Eindhoven, The Netherlands; r.l.p.d.d.jong@tue.nl (R.L.P.D.d.J.); m.breeuwer@tue.nl (M.B.); 2 Department of Surgery, University Medical Center Utrecht, 3584 CX Utrecht, The Netherlands; f.badaloni@umcutrecht.nl (F.B.); g.m.kuiper-2@umcutrecht.nl (G.M.K.); r.c.vanjaarsveld-3@umcutrecht.nl (R.C.v.J.); j.p.ruurda@umcutrecht.nl (J.P.R.); 3 Department of Electrical Engineering, Eindhoven University of Technology, 5612 AP Eindhoven, The Netherlands

**Keywords:** prototype networks, surgical scene understanding, semantic segmentation, surgical phase recognition, multitask learning, explainable AI, laparoscopic cholecystectomy, robot-assisted surgery, interpretable deep learning

## Abstract

Deep learning-based surgical vision systems achieve strong performance in semantic segmentation and phase recognition, but their black-box nature limits traceability in safety-critical clinical settings. Prototype-based networks offer an interpretable alternative by grounding predictions in learned visual exemplars, yet their suitability for surgical video understanding remains insufficiently characterized. We adapted a prototype-based architecture to two surgical datasets, laparoscopic cholecystectomy and robot-assisted minimally invasive esophagectomy (RAMIE), and benchmarked it against conventional baselines. We evaluated a segmentation-only setting, in which prototype size and capacity were ablated, and a multitask setting, in which three strategies for coupling prototype learning to semantic segmentation and surgical phase recognition were compared. Prototype-based models underperformed the conventional baselines across both tasks and datasets. In the segmentation-only setting, the selected prototype configurations achieved Dice scores of 72.23% on Cholecystectomy and 72.07% on RAMIE, compared with 74.35% and 74.02% for the corresponding conventional baselines, and showed weaker boundary agreement. In the multitask setting, the best prototype strategy recovered competitive segmentation performance but remained 6–12 F1 points below the conventional baseline for phase recognition. Qualitatively, prototype activation maps exposed intra-structure decompositions and contextual cues that are not directly available from black-box baselines. Prototype networks provide spatially traceable evidence for surgical scene understanding, but currently trade interpretability for reduced boundary precision and phase-recognition performance. These findings motivate future work on scene-level and temporally aware prototypes for explainable surgical AI.

## 1. Introduction

Deep learning has rapidly become the dominant paradigm in surgical vision, enabling strong performance in tasks such as semantic segmentation and surgical phase recognition. However, despite these advances, most state-of-the-art models remain largely opaque: they operate as black-box function approximators, making it difficult to understand the visual evidence driving their predictions or to verify their reliability in safety-critical surgical environments. This lack of transparency is particularly problematic in clinical settings, where interpretability and traceability are often as important as predictive accuracy.

Recent work has begun to address this limitation by introducing explainability into surgical vision systems. Kim et al. [1] proposed a concept-based explanation framework that maps surgical phase predictions to human-interpretable concepts, providing a more structured view of model reasoning. More recently, Cheng et al. [2] introduced a vision–language benchmarking framework integrating established XAI methods such as Grad-CAM [3] and CLEANN [4] to expose model attention patterns and causal relationships in predictions. While these approaches improve post-hoc interpretability, they do not fundamentally alter the underlying representation mechanism of the model, leaving predictions insufficiently traceable at the level of individual visual evidence.

A more principled alternative is prototype learning, in which predictions are grounded in learned visual exemplars rather than justified post hoc. This formulation enhances interpretability by identifying the prototypes and image regions that influence a prediction, while improving traceability by linking these prototypes to specific training examples. ProtoPNet [5] introduced this paradigm by representing each class with prototypical image parts drawn from the training data, thereby enabling case-based reasoning as an intrinsic part of the model. This was extended to dense prediction by ProtoSeg [6], demonstrating that prototype reasoning can be applied to semantic segmentation. Prototype-based methods have since gained considerable traction across medical image analysis, with applications spanning radiology [7,8,9], histopathology [10,11], and endoscopy [12], establishing prototype learning as a well-studied paradigm for interpretable medical vision [13]. However, the vast majority of these works address image-level classification, where a single global prediction suffices—a considerably simpler setting than surgical scene understanding, which demands both dense spatial localization of fine anatomical structures and holistic workflow-level phase recognition across temporally evolving video. Open challenges therefore remain in adapting prototype representations to such high-complexity settings.

In this work, we investigate prototype-based networks for surgical scene understanding, specifically semantic segmentation and surgical phase recognition in a multitask setting—a domain and problem formulation that, to the best of our knowledge, has not been previously addressed. We benchmark prototype-based models against conventional backbone–head baselines and multitask learning frameworks, evaluating both predictive performance and interpretability at the level of individual predictions, across two clinically distinct surgical datasets.

## 2. Materials and Methods

**Figure 1:**
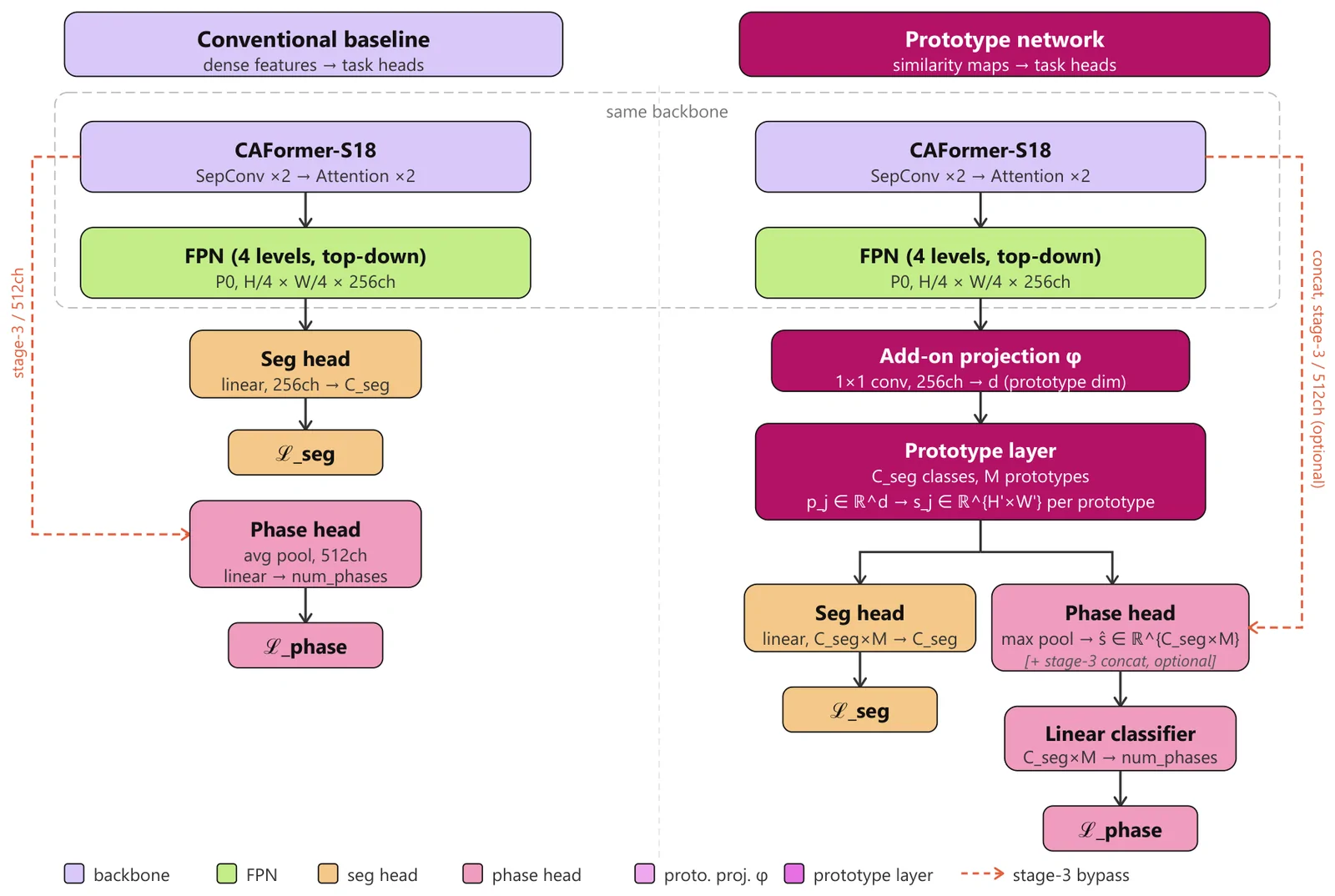
Overview of the conventional baseline and prototype network architectures investigated in this work.

Figure 1 provides an overview of the two frameworks investigated in this study. Both share the same feature extraction backbone, described in Section 2.1. The conventional baseline is detailed in Section 2.2, and the prototype-based architecture in Section 2.3.

### 2.1. Feature Extractor Backbone

A CAFormer-S18 backbone [14] pretrained on surgical data (SurgeNetXL) via self-supervised learning [15] is adopted for both frameworks. CAFormer-S18 follows the MetaFormer design: a four-stage hierarchical vision encoder combining local convolutional inductive biases with global transformer-style reasoning. The first two stages use depthwise separable convolutions as token mixers to capture local spatial structure; the last two stages use vanilla self-attention for long-range dependencies. Stages are separated by strided downsampling convolutions. For a 256×256 input, the four stages produce feature maps at spatial resolutions 64×64, 32×32, 16×16, and 8×8, with channel dimensions 64, 128, 320, and 512 respectively.

To obtain spatially rich features suitable for dense prediction, the four stage outputs are passed through a Feature Pyramid Network (FPN) [16]. The FPN first applies a lateral 1×1 convolution to each stage output to project all feature maps to a uniform channel width of 256. A top-down pathway then iteratively adds upsampled coarser features to finer ones, allowing every level to incorporate semantic context from deeper stages. A final 3×3 convolution is applied at each level to suppress aliasing artifacts from the upsampling. The result is four multi-scale feature maps, all with 256 channels, at spatial resolutions 64×64, 32×32, 16×16, and 8×8.

### 2.2. Conventional Model Architectures

The conventional baseline couples the shared backbone with two task-specific heads. For semantic segmentation, The finest-resolution FPN output is passed to a linear prediction head producing per-pixel class scores at 64×64 resolution. For surgical phase recognition, the final backbone stage features—prior to the FPN—are spatially collapsed via global average pooling and passed to a linear classifier. Both tasks are supervised with standard cross-entropy loss. This baseline contains no intermediate prototype space or similarity-based reasoning, and serves as a non-interpretable reference against which the trade-offs of the prototype network are evaluated.

### 2.3. Prototype-Based Model Architecture

#### 2.3.1. Prototype Construction

We employ the prototype-based architecture derived from ProtoPNet [5] and its dense prediction extension ProtoSeg [6]. The central principle of prototype learning is to represent each semantic class through a set of learnable prototypical feature patterns, such that model predictions can be attributed to image regions that resemble these learned reference concepts.

The proposed network consists of three main components: (i) a feature extraction backbone, (ii) a set of learnable prototype vectors, and (iii) task-specific prediction heads. Given an input image

$$x\in R^{H\times W\times C},$$

the backbone produces a latent feature tensor

$$f_{\theta }\left(x\right)\in R^{H^{\prime }\times W^{\prime }\times d_{f}},$$

where $H^{\prime }$ and $W^{\prime }$ denote the spatial dimensions after downsampling, $d_{f}$ is the backbone embedding dimension, and θ represents the backbone parameters. We use the finest-resolution FPN output described in Section 2.1 (64×64×256) as input to the prototype network, since it retains the highest spatial detail while still incorporating semantic context propagated from all scales. Each prototype, of size w×w, acts as a learned patch over this feature map.

To ensure compatibility between backbone features and prototype representations, a learnable projection ϕ(·) is applied when needed:

$$z=\phi \left(f_{\theta }\left(x\right)\right)\in R^{H^{\prime }\times W^{\prime }\times d},$$

where *d* is the prototype embedding dimension (d=128 in our experiments).

Each segmentation class $c\in C_{\mathrm{seg}}$ is assigned a fixed number *M* of prototypes, giving the complete prototype set

$$P={\left\{p_{j}\right\}}_{j=1}^{|C_{\mathrm{seg}}|\;M},$$

where each prototype is given by

$$p_{j}\in R^{w\times w\times d}.$$

Here, *w* denotes the prototype spatial size.

Each prototype is intended to encode a recurring local visual concept associated with its class. Examples include anatomical boundaries, characteristic tissue appearances, visually distinct subregions within the same anatomical structure, or representative surgical instrument features.

Let $z_{u,v}\in R^{w\times w\times d}$ denote the latent patch extracted from *z* at spatial location (u,v). The similarity between patch $z_{u,v}$ and prototype $p_{j}$ is computed using the ProtoPNet activation function:

$$s_{j}\left(u,v\right)=\log\left(\frac{{∥z_{u,v}-p_{j}∥}_{2}^{2}+1}{{∥z_{u,v}-p_{j}∥}_{2}^{2}+\varepsilon }\right),$$

where ε>0 is a small constant for numerical stability. Since this function is monotonically decreasing with respect to Euclidean distance, latent patches that are closer to a prototype produce larger activation values.

Applying this similarity computation densely over all spatial locations yields a set of prototype activation maps

$$s_{j}\in R^{H^{\prime }\times W^{\prime }},\;j=1,\ldots ,\left|P\right|.$$

The collection of activation maps ${\left\{s_{j}\right\}}_{j=1}^{\left|P\right|}$ forms an interpretable intermediate representation, encoding spatial correspondence between learned prototypes and image regions. This representation is subsequently used for downstream prediction tasks.

#### 2.3.2. Task Heads

**Segmentation Head**

For semantic segmentation, prototype activations are used to produce dense pixel-wise predictions. Let

$$s\left(u,v\right)={\left[s_{1}\left(u,v\right),\ldots ,s_{\left|P\right|}\left(u,v\right)\right]}^{⊤}$$

denote the prototype response vector at spatial location (u,v). These responses are mapped to per-pixel class scores, yielding the final segmentation output.

Since predictions are constructed from localized prototype similarities, each predicted region can be directly attributed to the prototypes that exhibit the strongest activation at the corresponding spatial locations.

**Surgical Phase Recognition Head**

For surgical phase recognition, a global image-level representation is required. Therefore, each prototype activation map is aggregated using global max pooling:

$${\hat{s}}_{j}=\max_{u,v}s_{j}\left(u,v\right),\;j=1,\ldots ,\left|P\right|.$$

This operation retains the strongest evidence of each prototype across the image, yielding the pooled representation

$$\hat{s}={\left[{\hat{s}}_{1},\ldots ,{\hat{s}}_{\left|P\right|}\right]}^{⊤}.$$

The resulting global prototype descriptor is used for surgical phase classification.

This design preserves interpretability by linking each phase prediction to the image regions that most strongly activate the corresponding prototypes.

#### 2.3.3. Training Procedure

Optimization of prototype-based networks is more challenging than conventional end-to-end learning, as the model must simultaneously learn discriminative latent representations, semantically meaningful prototype vectors, and stable task-specific classifiers. To improve convergence stability while preserving interpretability, training was conducted in four sequential stages following established prototype-learning frameworks [5,6].

**Stage 1: Warm-Up Optimization**

During the initial warm-up stage, the backbone parameters θ are frozen, while the prototype set P, projection module ϕ, and prediction heads are optimized:

$$\min_{P,\phi ,W,b}L.$$

Fixing the backbone stabilizes the latent feature space and allows prototypes to align with meaningful pre-trained representations before full network adaptation.

**Stage 2: Joint End-to-End Optimization**

After initialization, all model parameters are unfrozen and jointly optimized:

$$\min_{\theta ,P,\phi ,W,b}L.$$

This stage enables reciprocal refinement between feature learning and prototype learning. The backbone adapts to generate embeddings better suited for prototype matching, while prototypes become increasingly discriminative and semantically coherent.

**Stage 3: Prototype Projection (Push Step)**

Following gradient-based optimization, each prototype $p_{j}$ is projected onto the closest latent training patch:

$$p_{j}\leftarrow \arg\min_{z\in Z_{\mathrm{train}}}{∥z-p_{j}∥}_{2}^{2},$$

where $Z_{\mathrm{train}}$ denotes the set of latent feature patches extracted from the training set.

After projection, each prototype corresponds exactly to a real training-region representation rather than an unconstrained latent vector, thereby substantially improving interpretability.

**Stage 4: Final Classifier Fine-Tuning**

Because prototype projection alters prototype positions in feature space, the final classification heads may no longer be optimally calibrated. Therefore, the backbone and prototypes are frozen, while only the linear classifier parameters are optimized:

$$\min_{W,b}L.$$

This final stage recalibrates the mapping between prototype evidence and task outputs without modifying the learned interpretable prototype space.

#### 2.3.4. Loss Function

The optimization objective combines task-specific supervision with prototype regularization terms:

$$L_{\mathrm{total}}=L_{\mathrm{task}}+\lambda_{\mathrm{div}}L_{\mathrm{div}}+\lambda_{1}L_{1},$$

where $L_{\mathrm{task}}$ denotes the predictive loss, $L_{\mathrm{div}}$ is a prototype diversity constraint, and $L_{1}$ is an $L_{1}$ sparsity regularizer applied to classifier weights. The hyperparameters $\lambda_{\mathrm{div}}$ and $\lambda_{1}$ control the contribution of the regularization terms.

The task loss depends on the learning setting and is defined as

$$L_{\mathrm{task}}=\lambda_{\mathrm{seg}}L_{\mathrm{seg}}+\lambda_{\mathrm{phase}}L_{\mathrm{phase}},$$

where $L_{\mathrm{seg}}$ is the semantic segmentation cross-entropy loss and $L_{\mathrm{phase}}$ is the surgical phase recognition cross-entropy loss. In segmentation-only experiments, $\lambda_{\mathrm{phase}}=0$.

To prevent prototypes assigned to the same class from collapsing onto redundant representations, a diversity regularization term is introduced following ProtoSeg [6]:

$$L_{\mathrm{div}}=\sum_{c\in C}\sum_{\begin{array}{c} p_{i},p_{j}\in P_{c}\\ i<j \end{array}}\mathrm{sim}\left(p_{i},p_{j}\right),$$

where $P_{c}$ denotes the subset of prototypes assigned to class *c*, and sim(·,·) measures similarity between prototype vectors. Minimizing this term encourages prototypes of the same class to capture complementary visual evidence.

Following ProtoPNet [5], classifier sparsity is promoted through an additional $L_{1}$ penalty:

$$L_{1}=\sum_{c=1}^{C}\sum_{j:\;p_{j}\notin P_{c}}\left|w_{c,j}\right|,$$

where $w_{c,j}$ denotes the classifier weight linking prototype $p_{j}$ to class *c*. This regularization encourages class predictions to rely primarily on prototypes assigned to the corresponding class, thereby improving interpretability and reducing spurious cross-class prototype contributions.

#### 2.3.5. Multitask Learning Strategies for Surgical Phase Recognition

Surgical phase recognition and semantic segmentation impose qualitatively different representational demands. Segmentation requires dense, spatially precise features that resolve fine anatomical boundaries, whereas phase recognition requires a holistic, temporally stable summary of the operative scene. To investigate whether prototype-based representations can bridge these two objectives, three training strategies are evaluated, differing in when and how prototype learning is coupled to the phase supervision signal.

**Strategy A: Joint Multitask Prototype Learning.** All four training stages are executed under the full multitask objective, with segmentation and phase supervision applied simultaneously. Prototypes must therefore encode both spatially discriminative part-level concepts for dense prediction and globally discriminative scene-level concepts for workflow classification—an inherent representational tension that Strategy A is designed to diagnose.

**Strategy B: Segmentation-Guided Prototype Transfer.** The backbone, projection module, and prototypes are first learned under segmentation supervision and then frozen. During subsequent training, only the segmentation and phase classification heads remain trainable, treating prototype activation scores as fixed anatomical features. This isolates whether anatomy-driven prototypes alone constitute a sufficient representation for phase recognition, without access to the spatial and contextual features discarded by the prototype matching step.

**Strategy C: Segmentation-Guided Prototype Transfer with Dense Feature Augmentation.**

Strategy B is extended by augmenting the frozen prototype activation representation with global-average-pooled features from the last backbone stage before the phase classification head. As in Strategy B, the backbone, projection module, and prototypes remain frozen, while the segmentation and phase classification heads are trainable. The dense features may retain tissue context and inter-structure relationships that prototype matching discards. Strategy C thus evaluates whether interpretability can be preserved while recovering the representational capacity lost through the prototype bottleneck.

Comparing these three strategies provides a controlled decomposition of two interacting questions: (i) whether prototype representations should be jointly adapted for phase recognition or kept anchored to their segmentation-derived semantics, and (ii) whether the prototype activation vector alone is a sufficient intermediate representation for downstream workflow classification, or whether it must be augmented with dense contextual features to achieve competitive performance.

### 2.4. Dataset

Both datasets used in this study share a common structure: surgical videos are annotated with phase labels throughout, while a subset of frames or sequences are additionally annotated with scene segmentation masks covering surgical instruments and anatomical structures. The characteristics of each dataset are summarized in the following subsections.

#### 2.4.1. Laparoscopic Cholecystectomy

Segmentation annotations are taken from CholecSeg8k [17] and phase annotations from Cholec80 [18]. The segmentation labels cover 13 classes: five anatomical structures (abdominal wall, liver, gastrointestinal tract, fat, and connective tissue), three vascular and connective structures (cystic duct, hepatic vein, and liver ligament), two instrument types (laparoscopic hook electrocautery and grasper), one organ target (gallbladder), one fluid class (blood), and a background class. Phase annotations span 7 surgical phases: preparation, calot triangle dissection, clipping and cutting, gallbladder dissection, gallbladder packaging, cleaning and coagulation, and gallbladder retraction.

Data are split by video index: videos 1–32 for training (71,000 phase-annotated frames, 5360 segmentation-annotated frames), videos 33–40 for validation (15,304 phase, 720 segmentation), and videos 41–80 for testing (98,194 phase, 2000 segmentation frames).

#### 2.4.2. Robot-Assisted Minimally Invasive Esophagectomy (RAMIE)

The Robot-Assisted Minimally Invasive Esophagectomy (RAMIE) dataset is an in-house dataset from the University Medical Center (UMC) Utrecht, the Netherlands. Surgical recordings were prospectively collected from patients undergoing transthoracic RAMIE for esophageal cancer between January 2018 and July 2021; the only exclusion criterion was the absence of a thoracic recording. For both the segmentation and phase recognition datasets, procedures were randomly selected for annotation. The segmentation dataset extends that of de Jong et al. [19] and comprises 1504 annotated frames from 53 procedures across 12 foreground classes, including four instrument types (cadiere forceps, cautery hook, laparoscopic suction, and vessel sealer) and eight anatomical structures (airways, aorta, azygos vein, vagal nerves, pericardium, right lung, recurrent laryngeal nerve, and thoracic duct). The phase recognition dataset comprises annotations of 13 surgical phases across 279,251 frames from 38 procedures, extending the benchmark dataset of Li et al. [20]. The annotation protocol has been described in [21].

Data are split at the patient level into training (38 patients, 1025 segmentation frames; 21 patients, 162,943 phase frames), validation (5 patients, 137 segmentation frames; 6 patients, 41,527 phase frames), and test (10 patients, 342 segmentation frames; 11 patients, 74,781 phase frames) sets. Patients appearing in both subsets are assigned to the same partition to prevent cross-task data leakage.

### 2.5. Evaluation Metrics

#### 2.5.1. Segmentation Evaluation Metrics

Segmentation performance is evaluated using the Dice similarity coefficient and the 95th-percentile Hausdorff distance (HD95). Dice measures volumetric overlap between predicted and ground-truth masks, ranging from 0 to 1, and is computed by pooling all predictions across the test set into a single global confusion matrix per class; the reported mean and standard deviation are computed across foreground classes present in the test set. HD95 measures boundary agreement in pixels, with lower values indicating closer contour alignment; it is computed per class per image, averaged across images to obtain a per-class score, and the reported mean and standard deviation are computed across foreground classes.

#### 2.5.2. Phase Evaluation Metrics

Surgical phase recognition is evaluated using frame-level accuracy and macro-averaged F1 score. Accuracy measures the fraction of correctly classified frames across all phases. For each phase class, F1 is the harmonic mean of precision and recall; macro F1 averages these per-class scores equally across all phase classes. Accuracy is reported with its standard deviation across patients; macro F1 is reported with its standard deviation across phase classes.

### 2.6. Prototype Activation Evaluation

To quantify whether learned prototypes provide class-specific and complementary spatial evidence, we designed two metrics: prototype activation purity and within-class prototype redundancy. Each prototype $p_{j}$ was first assigned to a semantic class $c_{j}$ using the prototype-class identity matrix. For each test image in which class $c_{j}$ was present, we kept only the prototype’s top-activation region, defined as the highest ρ fraction of spatial activation values. In our experiments, ρ=0.05, corresponding to the top 5% most activated locations:

$$B_{j}^{\left(n\right)}\left(u,v\right)=1\left[s_{j}^{\left(n\right)}\left(u,v\right)\;\mathrm{belongs}\;\mathrm{to}\;\mathrm{the}\;\mathrm{top}\;\rho \;\mathrm{fraction}\;\mathrm{of}\;\mathrm{activation}\;\mathrm{values}\right].$$

Here, *n* indexes the test image, (u,v) indexes a spatial location on the prototype activation map, $s_{j}^{\left(n\right)}\left(u,v\right)$ is the activation value of prototype $p_{j}$ at that location, and 1[·] is the indicator function. Therefore, $B_{j}^{\left(n\right)}\left(u,v\right)$ is a binary mask that equals 1 for selected top-activation locations and 0 elsewhere.

Purity measures whether a prototype’s strongest responses fall inside the class it is supposed to represent. For prototype $p_{j}$, the purity score is

$$P_{j}=\frac{\sum_{n\in I_{c_{j}}}\sum_{u,v}B_{j}^{\left(n\right)}\left(u,v\right)G_{c_{j}}^{\left(n\right)}\left(u,v\right)}{\sum_{n\in I_{c_{j}}}\sum_{u,v}B_{j}^{\left(n\right)}\left(u,v\right)},$$

where $I_{c_{j}}$ is the set of test images containing class $c_{j}$, and $G_{c_{j}}^{\left(n\right)}\left(u,v\right)$ is the resized ground-truth mask for class $c_{j}$ at location (u,v). The numerator counts selected prototype activations that overlap the assigned class, while the denominator counts all selected prototype activations. Thus, a high purity score means that the prototype mainly activates on the correct anatomy or instrument, whereas a low score means that it often activates on background, nearby tissue, tools, or other context.

Redundancy measures whether different prototypes assigned to the same class focus on the same spatial evidence. For every pair of prototypes $i,j\in P_{c}$ assigned to class *c*, we computed the intersection-over-union of their top-activation regions:

$$R_{i,j}^{\left(n\right)}=\frac{\left|B_{i}^{\left(n\right)}\cap B_{j}^{\left(n\right)}\right|}{\left|B_{i}^{\left(n\right)}\cup B_{j}^{\left(n\right)}\right|}.$$

The final redundancy value for a prototype pair is obtained by averaging $R_{i,j}^{\left(n\right)}$ across class-present test images. High redundancy means that same-class prototypes repeatedly focus on similar regions, whereas low redundancy means that they capture different regions or appearances. Low redundancy is most meaningful when purity is also high; otherwise, the prototypes may simply be attending to different irrelevant regions.

### 2.7. Implementation Details

For both the conventional baseline and the prototype-based network, the segmentation and phase-recognition loss weights are set to $\lambda_{\mathrm{seg}}=1$ and $\lambda_{\mathrm{phase}}=2$, respectively. The phase-recognition task uses a batch size four times larger than that of segmentation because more phase-labeled data are available. Nevertheless, the segmentation loss converges more rapidly during joint training; therefore, the phase-recognition loss is assigned a larger weight to balance the learning progress of the two tasks. For the prototype-based network, additional regularization terms are applied with $\lambda_{1}=1\times 10^{-4}$ and $\lambda_{\mathrm{div}}=0.25$. All experiments are conducted on a single NVIDIA H100 GPU using the Adam optimizer, with segmentation and phase-recognition batch sizes of 32 and 128, respectively. Validation is performed every 200 training steps, and early stopping with a patience of 10 validation evaluations is used to prevent overfitting. When the corresponding modules are trainable, the learning rates are set as follows: $5\times 10^{-5}$ for the backbone, $2.5\times 10^{-4}$ for both the add-on projection module and prototype vectors, $5\times 10^{-6}$ for the segmentation head, and $1\times 10^{-5}$ for the phase-recognition head. Details of the training procedure are provided in Section 2.3.3. All images are resized to 256×256 pixels. Patch accuracy measures the proportion of spatial locations assigned the correct segmentation class, whereas image accuracy measures the proportion of images assigned the correct phase label. Early stopping is based on patch accuracy for segmentation-only training and on a combined patch- and image-accuracy criterion for multitask training.

## 3. Experiments and Results

### 3.1. Prototype Network Configuration Studies

We conduct two controlled ablation studies to determine the optimal prototype configuration for the segmentation task. In both studies, the prototype-based network is evaluated against a conventional segmentation baseline trained under identical conditions.

**Prototype size.** The prototype size controls the spatial extent of the feature patch each prototype represents. A 1×1 prototype captures purely local, point-wise features, while larger sizes encode broader spatial context. We evaluate sizes 1×1, 3×3, and 5×5 with the number of prototypes fixed at 12.

**Number of prototypes.** The number of prototypes determines the capacity of the prototype library to represent the diversity of visual concepts present in the training data. Too few prototypes risk underfitting the variety of anatomical appearances, while too many may introduce redundancy or hinder training convergence. We evaluate 8, 12, and 20 prototypes using the best-performing prototype size from the previous experiment.

Table 1 and Table 2 show that the optimal prototype size differs by dataset. On Cholecystectomy, 1×1 prototypes perform best (81.97% Dice, 36.69 HD95), whereas RAMIE favors 3×3 prototypes (69.83% Dice, 36.26 HD95). In both datasets, 5×5 prototypes perform substantially worse, suggesting that overly broad prototype patches blur the fine boundaries needed for surgical segmentation.

The capacity ablation shows a consistent optimum at 12 prototypes per class. Increasing the prototype count from 8 to 12 improves Dice on both Cholecystectomy (74.36% to 81.97%) and RAMIE (67.89% to 69.83%), whereas increasing it further to 20 prototypes reduces Dice and worsens HD95. The validation-selected configurations are therefore 1×1 with 12 prototypes for Cholecystectomy and 3×3 with 12 prototypes for RAMIE.

Table 3 reports held-out test performance for these validation-selected configurations. The prototype models achieve reasonable segmentation performance but remain below the conventional baselines: Dice decreases from 74.35% to 72.23% on Cholecystectomy and from 74.02% to 72.07% on RAMIE, while HD95 increases from 30.92 to 43.67 and from 25.10 to 32.82, respectively.

### 3.2. Qualitative Analysis of Prototype Activations

For both datasets, the qualitative activation analysis uses the validation-selected best-performing prototype configuration identified above: 1×1 prototypes with 12 prototypes per class for Cholecystectomy, and 3×3 prototypes with 12 prototypes per class for RAMIE.

For each test image, each prototype is assigned an image-level activation score by averaging its activation map over all spatial locations:

$${\bar{s}}_{j}=\frac{1}{HW}\sum_{u=1}^{H}\sum_{v=1}^{W}s_{j}\left(u,v\right),\;j=1,\ldots ,\left|P\right|,$$

where *H* and *W* denote the spatial dimensions of the activation map. Prototypes are then ranked according to ${\bar{s}}_{j}$, and the top-*K* prototypes with the highest average activation scores are selected for visualization. The corresponding activation maps are overlaid on the input image to highlight the spatial regions that contribute most strongly to each prototype response.

**Figure 2:**
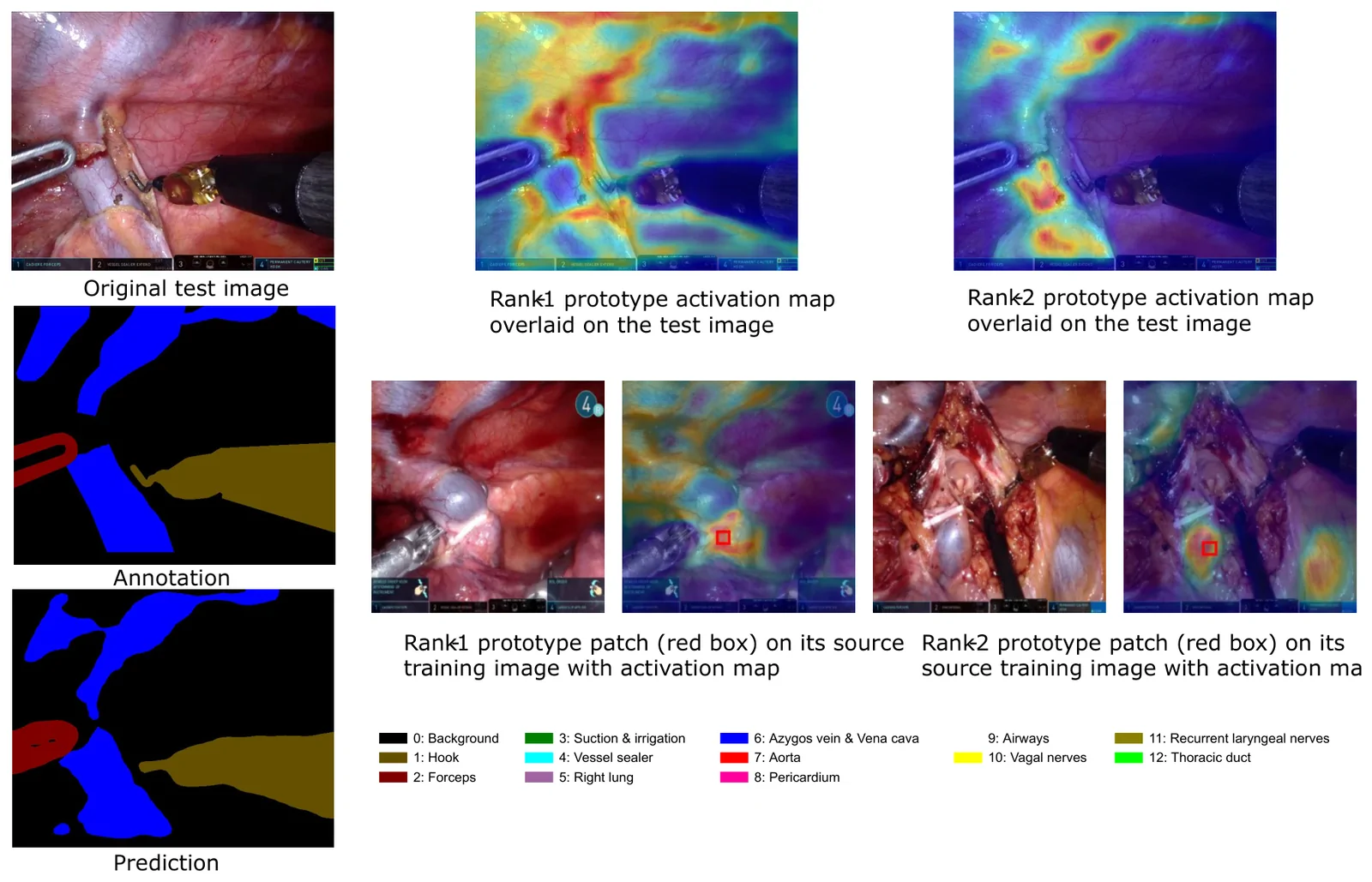
Prototype-based segmentation and activation behavior on a representative RAMIE frame with the azygos vein and vena cava (class 6, blue). The selected prototypes highlight distinct vessel subregions, illustrating intra-class anatomical decomposition.

**Figure 3:**
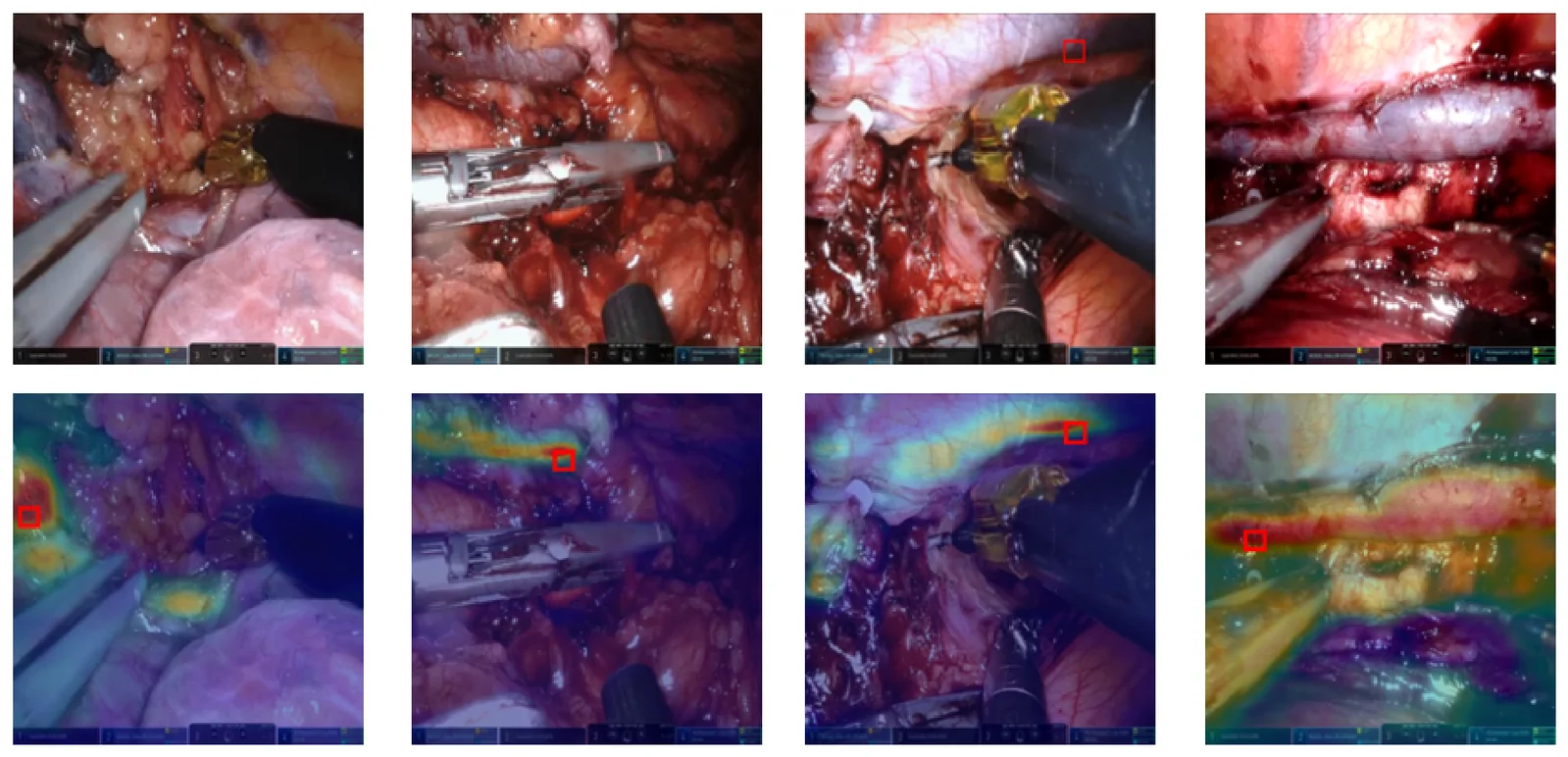
Additional learned RAMIE prototypes for the azygos vein class. Red boxes indicate projected training patches, and activation overlays show that different prototypes capture recurring vessel appearances.

Figure 2 illustrates a representative RAMIE example for the azygos vein class. The highest-ranked prototype responds primarily to branching portions of the vessel, whereas the second-ranked prototype focuses on the central connective segment. Figure 3 shows additional learned prototypes for this class, indicating that the prototype library captures multiple recurring appearances of the azygos vein across training frames.

**Figure 4:**
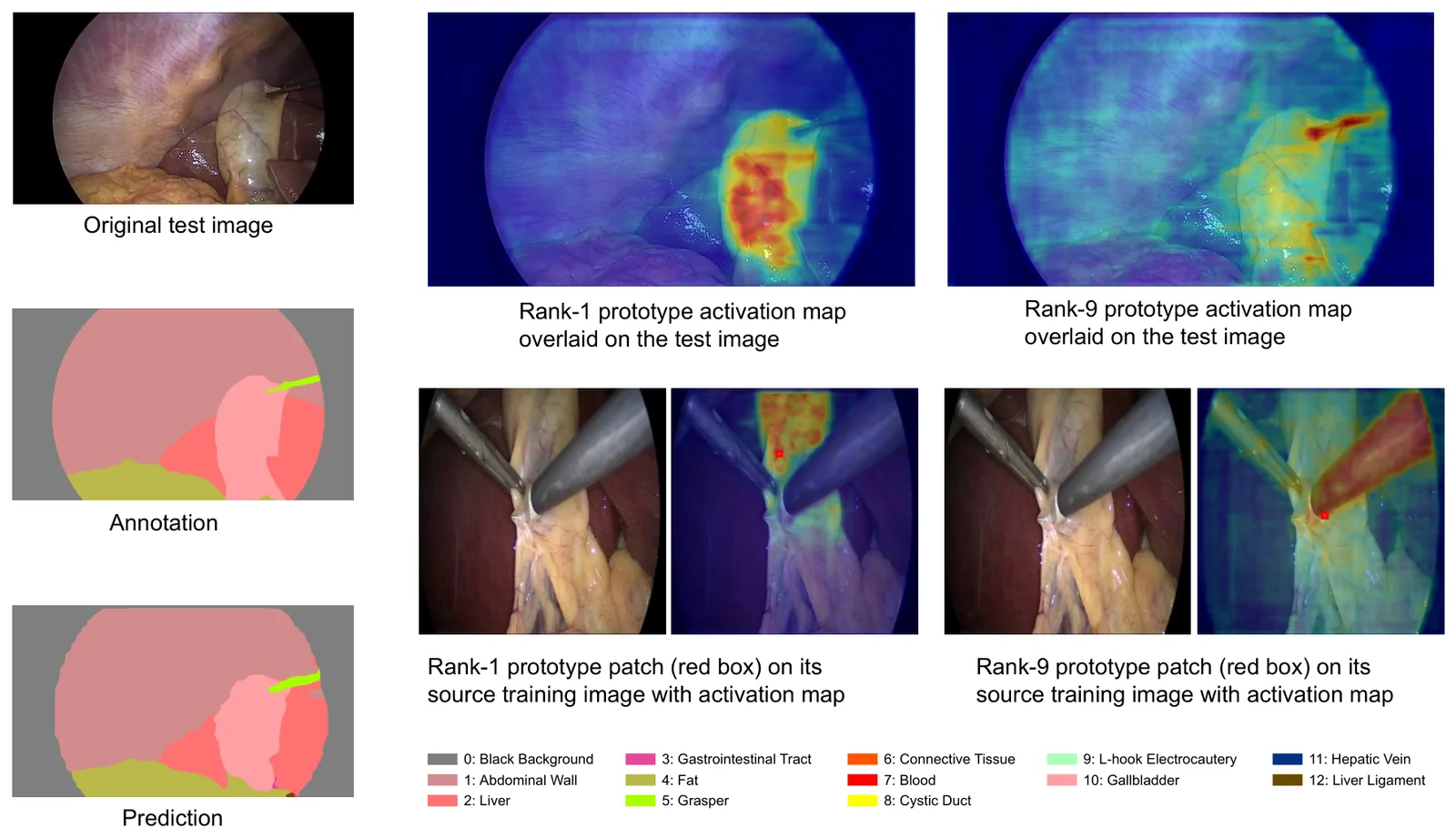
Prototype-based segmentation and activation behavior on a representative Cholecystectomy frame depicting the gallbladder (class 10, light pink). The selected prototypes localize the gallbladder region while also exposing a tool-related contextual cue associated with this anatomical class.

Figure 4 shows a representative Cholecystectomy example for the gallbladder class. The highest-ranked prototype localizes to the central body of the gallbladder, while a lower-ranked selected prototype responds to a tool interacting with the organ. Because the corresponding source patch also contains tool-related evidence, this example shows how a prototype assigned to an anatomical class can expose a correlated contextual cue rather than a purely anatomical concept. This visibility is useful because the prototype framework makes the association inspectable through both the training source patch and the activation region on the test image.

The predicted segmentation masks in Figure 2 and Figure 4 generally correctly localize the target structures but show less clearly delineated boundaries than the corresponding ground-truth annotations.

**Figure 5:**
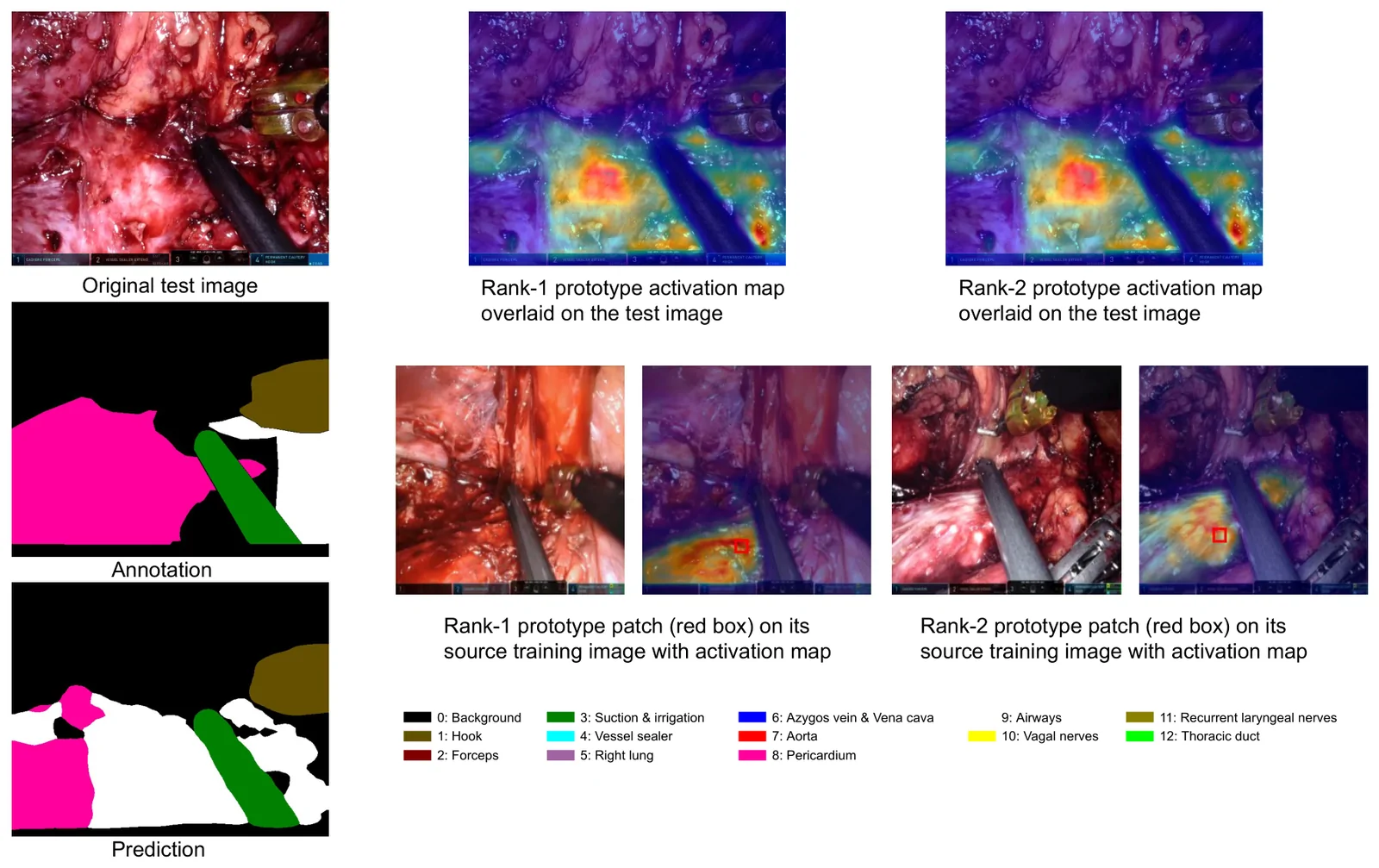
Failure example for RAMIE airway segmentation. The two highest-ranked airway prototypes produce similar activation maps and incorrectly respond to the pericardium, despite visually different projected training patches.

Figure 5 shows a failing RAMIE example for the Airways class. The two highest-ranked airway prototypes produce almost identical activation maps and both incorrectly activate the pericardium as airway, despite originating from visually different projected training patches. This failure illustrates that diversity among source patches does not necessarily translate into complementary activation behavior at test time, especially for classes with limited training data.

### 3.3. Prototype Activation Analysis

**Figure 6:**
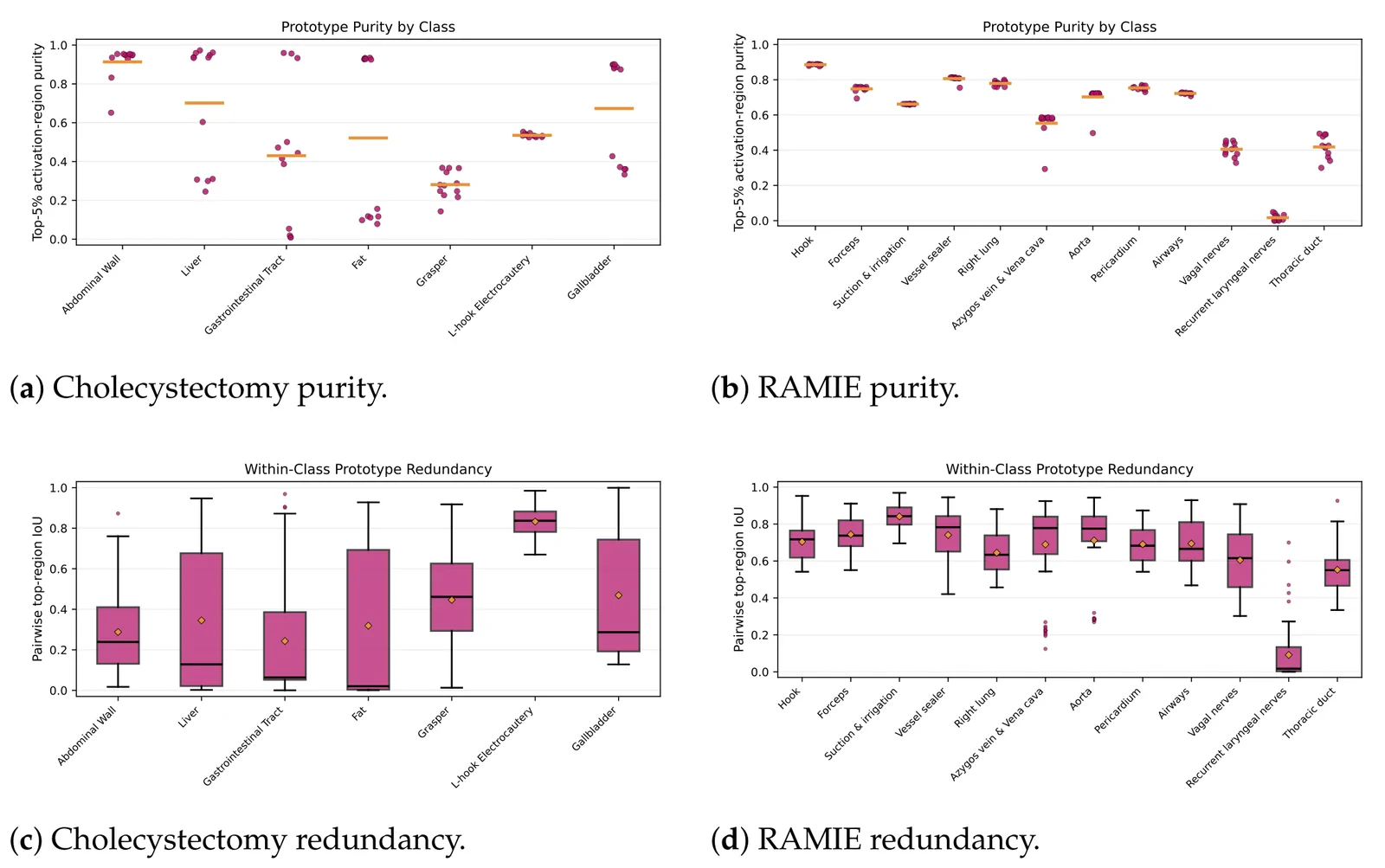
Class-wise prototype activation purity and within-class redundancy on the Cholecystectomy (**a**,**c**) and RAMIE datasets (**b**,**d**). Purity is defined as the fraction of a prototype’s top 5% activated patches that fall within its assigned ground-truth class. Each dot represents one prototype, with higher values indicating more class-specific localization. Redundancy is the average overlap between the top 5% activation regions of same-class prototype pairs; higher values indicate that prototypes assigned to the same class rely on more similar spatial evidence. Orange horizontal strips in the purity panels and orange markers in the redundancy panels denote class means.

Figure 6 shows that prototype activation quality differs substantially across datasets and classes. In Cholecystectomy, abdominal-wall prototypes achieve high mean purity, likely because the structure has a large size and visually distinctive appearance that facilitates the learning of class-specific representations. Their relatively low redundancy further suggests that different prototypes capture distinct regions or appearances of the abdominal wall. Liver, gastrointestinal tract, fat, and gallbladder contain both high-purity anatomical prototypes and lower-purity prototypes that may encode contextual cues. In contrast, grasper and L-hook electrocautery prototypes show relatively low purity. This may partly reflect their small spatial extent: because purity is evaluated using a fixed top 5% activation region, classes occupying less than this proportion of the image are inherently penalized when selected activations extend beyond the annotated mask.

RAMIE exhibits a different class-wise pattern. Several instruments and visually distinctive structures, including the hook, vessel sealer, and right lung, contain comparatively high-purity prototypes. However, these classes also show generally high redundancy, indicating that same-class prototypes often rely on similar spatial evidence. The azygos vein and aorta classes achieve moderate-to-high purity while containing several low-redundancy outliers, suggesting that a subset of prototypes may capture distinct anatomical subregions or appearances. By contrast, the fine neural structures are represented less effectively. Vagal-nerve prototypes show reduced purity, while recurrent-laryngeal-nerve prototypes exhibit minimal overlap with the assigned class. The combination of low purity and low redundancy suggests that the latent feature space did not learn a stable, class-discriminative representation for this small and visually subtle structure.

### 3.4. Multitask Comparison

Table 4 and Table 5 report quantitative results for the three multitask learning strategies across both datasets. In all configurations, conventional multitask learning outperforms every prototype-based strategy on phase recognition, and prototype strategies recover segmentation performance only partially and inconsistently across datasets.

**Strategy A** yields the weakest overall performance, with notable degradation on both tasks relative to conventional multitask learning. When a shared prototype layer must simultaneously serve dense segmentation and coarse phase objectives, neither task receives well-suited representations. Degradation is more pronounced on phase recognition than on segmentation—on Cholecystectomy, F1 drops by 13.8 points (74.30 to 60.51) versus a 4.1 point Dice drop, and on RAMIE, F1 drops by 11.5 points (74.99 to 63.53) versus a 4.1 point Dice drop. This asymmetry is consistent with the per-pixel segmentation loss dominating gradient updates, crowding out phase-discriminative signal.

**Strategy B** recovers segmentation performance toward the single-task prototype baseline on the Cholecystectomy dataset (72.27% vs. 72.23%), suggesting that freezing the learned prototype representation while allowing the segmentation head to remain trainable largely preserves its spatial semantics on that domain. However, this recovery does not generalize to RAMIE, where Strategy B Dice (68.37%) remains 3.7 points below the single-task prototype result (72.07%), indicating that prototype representations learned on one training regime may not transfer robustly under multitask fine-tuning across datasets. Phase recognition remains substantially below the conventional baseline in both cases, exposing the limitation of the prototype bottleneck: part-level activation vectors discard the contextual and relational information that phase recognition requires—relative spatial configuration, tissue state, and inter-structure relationships are not encoded in prototype similarity scores.

**Strategy C** consistently achieves the best phase recognition and segmentation performance among the three prototype strategies on both datasets. Augmenting the frozen prototype activations with global-average-pooled features from the last-stage of the backbone map restores access to dense spatial features discarded by the prototype matching step, partially recovering phase recognition capacity (F1: 62.93 on Cholecystectomy, 68.94 on RAMIE). Segmentation also improves marginally over Strategy B on both datasets (73.72 vs. 72.27 Dice on Cholecystectomy; 70.11 vs. 68.37 on RAMIE), reflecting differences in the jointly optimized task heads while the underlying prototype representation remains fixed. Strategy C therefore represents the most favorable trade-off: interpretable anatomy-grounded prototypes are preserved for segmentation while partially recovering phase recognition via dense feature bypass.

For HD95, Strategy C decreases the Cholecystectomy value from 43.67 in the single-task prototype model to 34.42 in the multitask setting, slightly below the conventional multitask baseline value of 34.98. On RAMIE, Strategy C obtains an HD95 of 33.52, close to the single-task prototype value of 32.82 and above the conventional multitask baseline value of 25.56.

## 4. Discussion

### 4.1. Prototype Interpretability and Segmentation Trade-Offs

This study shows that prototype networks provide spatially traceable explanations for surgical segmentation, but at the cost of reduced quantitative performance. This trade-off may partly reflect a mismatch between prototype learning and surgical anatomy. Prototype methods are most effective when visual concepts can be decomposed into stable, recurring patterns, whereas surgical structures are often continuous, deformable, and variable in scale and appearance across patients and viewpoints.

Despite these challenges, the qualitative analyses show that individual prototypes can represent recognizable anatomical subregions as well as meaningful contextual cues, such as instruments interacting with specific tissues. The purity and redundancy analyses further demonstrate that interpretability is class-dependent. Some prototypes localize accurately to their assigned structures but repeatedly attend to similar regions, indicating limited diversity. Others show lower purity because they capture correlated contextual information. The presence of several low-purity prototypes within a class should therefore not automatically be interpreted as failure, as such prototypes may still reveal meaningful associations used by the model. However, consistently low purity combined with spatially inconsistent activation provides stronger evidence that the network has failed to learn a stable class-specific representation.

The quantitative results and qualitative examples also show that prototype models have higher HD95 values than conventional baselines and weak contour alignment. A likely explanation is that prototype matching relies on similarity to learned feature patches, producing broader and more diffuse activation patterns than direct dense-feature decoding. This can preserve interpretable evidence while reducing boundary precision.

The configuration studies further indicate that prototype granularity must be matched to the dataset. The best validation setting used 1×1 prototypes for Cholecystectomy and 3×3 prototypes for RAMIE, whereas 5×5 prototypes performed poorly on both datasets. Overly large prototypes may blur fine anatomical boundaries, while highly local prototypes may be insufficient for structures that require broader spatial context. Similarly, 12 prototypes per class outperformed both 8 and 20, suggesting an intermediate-capacity regime that captures recurring appearances without introducing excessive redundancy or optimization difficulty.

### 4.2. Prototype Bottlenecks in Multitask Learning

The multitask experiments indicate that prototype representations are better suited to structured anatomical decomposition than to phase recognition when used as the only intermediate representation. Strategy A performed worst among the prototype strategies, suggesting that a single shared prototype layer is difficult to optimize when it must support both pixel-level segmentation and image-level phase classification. This tension is most visible in phase recognition, where the drop relative to the conventional multitask baseline was larger than the corresponding drop in Dice on both datasets.

Strategy B and Strategy C clarify the role of the prototype bottleneck. Freezing segmentation-trained prototypes in Strategy B preserved segmentation performance on Cholecystectomy, but phase recognition remained substantially below the conventional baseline. This suggests that averaged prototype activations alone discard contextual information needed for workflow reasoning, including spatial configuration, tissue state, and inter-structure relationships. Strategy C partially addressed this limitation by adding dense backbone features alongside the prototype activations. It achieved the strongest prototype-based results on both datasets, making it the most favorable trade-off among the evaluated prototype strategies: anatomy-grounded prototype activations remain available for segmentation interpretability, while dense features recover part of the phase-recognition capacity lost through the prototype bottleneck. The remaining F1 gap between Strategy C and the conventional multitask baseline, however, shows that this dense feature bypass does not fully restore the information used by the black-box model.

### 4.3. Dataset-Specific Effects

Strategy C affected boundary accuracy differently across datasets. In Cholecystectomy, HD95 improved relative to the single-task prototype model and approached the conventional multitask baseline, possibly because anatomical structures and instruments occur in more consistent, phase-specific spatial configurations. In RAMIE, HD95 remained above the conventional baseline. Its more closely zoomed views often show structures only partially, while tissue mobilization and viewpoint variation further increase appearance variability. These factors may reduce the extent to which phase context can improve boundary localization.

The same imaging characteristics may also help explain the generally higher prototype redundancy in RAMIE than in Cholecystectomy. Because structures are observed across variable and incomplete views, distinct recurring subregions may be difficult to identify consistently. The model may therefore converge on the limited visual characteristics shared across training images, causing multiple same-class prototypes to rely on similar evidence. This may explain the high redundancy observed in classes with moderate-to-high purity.

### 4.4. Limitations and Future Work

Several limitations arise from the current formulation. First, prototypes are learned for individual anatomical structures and instruments rather than for higher-order scene configurations. Scene-level prototypes could better support workflow reasoning by representing relationships among anatomy, instruments, and tissue state. Second, both segmentation and phase recognition are performed at the frame level. Incorporating temporal information through recurrent models, temporal attention, or sequences of prototype activations may improve phase recognition while preserving interpretability. Third, prototype quality depends strongly on the underlying latent representation. If the backbone does not adequately separate relevant visual concepts, prototype projection may associate prototypes with misleading regions and propagate these errors to downstream predictions. Although this study used a surgically pretrained encoder to provide a stronger initialization, future advances in surgical representation learning may further improve the stability, specificity, and effectiveness of learned prototypes.

## 5. Conclusions

This work evaluated prototype networks against conventional baselines on two surgical vision datasets for semantic segmentation and phase recognition. Prototype-based architectures provided reasonably interpretable, spatially traceable evidence through learned prototype vectors and their corresponding activation maps, but underperformed conventional baselines quantitatively, particularly for boundary-sensitive segmentation and phase recognition. Among the evaluated multitask strategies, augmenting frozen prototype activations with dense backbone features produced the strongest prototype-based performance, showing that interpretability can be partly preserved while recovering some task performance.

Overall, prototype-based architectures offer a principled but currently imperfect path toward interpretable surgical scene understanding. The appropriate balance between transparency and quantitative performance is likely to depend on the clinical application. In some settings, reliable identification of relevant structures may be more important than highly precise contour delineation, although systems that achieve both remain the ultimate goal. Future work should therefore investigate scene-level and temporally aware prototypes, stronger surgical feature representations, and application-specific evaluation. Advancing these directions is essential for developing surgical AI systems that are not only more interpretable, but also quantitatively robust and clinically meaningful.

## Figures and Tables

**Table 1:** Validation-set ablation of prototype configuration in a segmentation-only setting on the Cholecystectomy dataset. Prototype size experiments are conducted with 12 prototypes; number-of-prototypes experiments use the best-performing prototype size. Bold values indicate the best setting within each ablation group, using higher Dice and lower HD95 as the selection criteria.

		Cholecystectomy	
Group	Setting	Dice (%)	HD95
Prototype size	1×1	81.97±8.65	36.69±22.25
	3×3	77.81±10.04	41.18±19.72
	5×5	40.48±38.01	115.69±32.83
No. of prototypes	8	74.36±9.55	37.82±24.75
	12	81.97±8.65	36.69±22.25
	20	72.16±12.10	40.84±26.85

**Table 2:** Validation-set ablation of prototype configuration in a segmentation-only setting on the RAMIE dataset. Prototype size experiments are conducted with 12 prototypes; number-of-prototypes experiments use the best-performing prototype size. Bold values indicate the best setting within each ablation group, using higher Dice and lower HD95 as the selection criteria.

		RAMIE	
Group	Setting	Dice (%)	HD95
Prototype size	1×1	65.86±21.62	39.00±21.63
	3×3	69.83±19.95	36.26±19.09
	5×5	44.09±30.17	74.85±46.91
No. of prototypes	8	67.89±20.77	40.45±22.17
	12	69.83±19.95	36.26±19.09
	20	67.68±21.38	41.11±23.10

**Table 3:** Held-out test performance of the validation-selected segmentation-only prototype configurations and corresponding conventional segmentation baselines. Bold values indicate the better result within each dataset, using higher Dice and lower HD95 as the comparison criteria.

Dataset	Model	Dice (%)	HD95
Cholecystectomy	Conventional segmentation	74.35±16.82	301.92±10.51
	Validation-selected prototype (1×1, 12 prototypes)	72.23±20.96	43.67±27.11
RAMIE	Conventional segmentation	74.02±21.34	25.10±12.52
	Validation-selected prototype (3×3, 12 prototypes)	72.07±23.35	32.82±14.48

**Table 4:** Comparison of conventional and prototype-based approaches in a multitask learning setting on CholecSeg8k. Bold values indicate the best-performing prototype-based strategy among Strategies A–C for each metric.

Setting	Dice (%)	HD95	Acc. (%)	F1 (%)
Conventional multitask learning	71.85±28.22	34.98±26.48	83.29±7.16	74.30±9.94
Strategy A	67.72±25.65	51.12±25.67	74.87±8.12	60.51±12.68
Strategy B	72.27±19.81	36.29±14.75	73.50±8.12	59.39±13.32
Strategy C	73.72±19.59	34.42±13.07	75.49±8.56	62.93±12.10

**Table 5:** Comparison of conventional and prototype-based approaches in a multitask learning setting on RAMIE. Bold values indicate the best-performing prototype-based strategy among Strategies A–C for each metric.

Setting	Dice (%)	HD95	Acc. (%)	F1 (%)
Conventional multitask learning	71.77±22.92	25.56±12.16	80.43±4.81	74.99±10.00
Strategy A	67.72±24.59	35.85±19.98	71.15±6.98	63.53±15.54
Strategy B	68.37±25.05	43.73±32.34	69.81±8.11	61.51±16.68
Strategy C	70.11±23.51	33.52±15.22	76.03±5.43	68.94±13.41

## Data Availability

The RAMIE dataset presented in this article is not publicly available due to technical limitations. The CholecSeg8k and Cholec80 datasets used for the cholecystectomy experiments are publicly available and can be accessed on https://doi.org/10.48550/arXiv.2012.12453 and https://doi.org/10.1109/TMI.2016.2593957 (accessed on 4 August 2026) reference number [17,18].

## Abbreviations

The following abbreviations are used in this manuscript:

RAMIE	Robot-assisted minimally invasive esophagectomy
FPN	Feature Pyramid Network
HD95	95th-percentile Hausdorff Distance
XAI	Explainable Artificial Intelligence
